# Completeness of Follow-Up Determines Validity of Study Findings: Results of a Prospective Repeated Measures Cohort Study

**DOI:** 10.1371/journal.pone.0140817

**Published:** 2015-10-15

**Authors:** Regula S. von Allmen, Salome Weiss, Hendrik T. Tevaearai, Christoph Kuemmerli, Christian Tinner, Thierry P. Carrel, Juerg Schmidli, Florian Dick

**Affiliations:** 1 Department of Vascular Surgery, Kantonsspital St. Gallen, 9007 St. Gallen, Switzerland; 2 Department of Cardiovascular Surgery, University Hospital and University of Bern, 3010 Bern, Switzerland; School of Medicine, Fu Jen Catholic University, TAIWAN

## Abstract

**Background:**

Current reporting guidelines do not call for standardised declaration of follow-up completeness, although study validity depends on the representativeness of measured outcomes. The *Follow-Up Index* (FUI) describes follow-up completeness at a given study end date as ratio between the investigated and the potential follow-up period. The association between FUI and the accuracy of survival-estimates was investigated.

**Methods:**

FUI and Kaplan-Meier estimates were calculated twice for 1207 consecutive patients undergoing aortic repair during an 11-year period: in a scenario A the population’s clinical routine follow-up data (available from a prospective registry) was analysed conventionally. For the control scenario B, an independent survey was completed at the predefined study end. To determine the relation between FUI and the accuracy of study findings, discrepancies between scenarios regarding FUI, follow-up duration and cumulative survival-estimates were evaluated using multivariate analyses.

**Results:**

Scenario A noted 89 deaths (7.4%) during a mean considered follow-up of 30±28months. Scenario B, although analysing the same study period, detected 304 deaths (25.2%, *P*<0.001) as it scrutinized the complete follow-up period (49±32months). FUI (0.57±0.35 versus 1.00±0, *P*<0.001) and cumulative survival estimates (78.7% versus 50.7%, *P*<0.001) differed significantly between scenarios, suggesting that incomplete follow-up information led to underestimation of mortality. Degree of follow-up completeness (i.e. FUI-quartiles and FUI-intervals) correlated directly with accuracy of study findings: underestimation of long-term mortality increased almost linearly by 30% with every 0.1 drop in FUI (adjusted HR 1.30; 95%-CI 1.24;1.36, *P*<0.001).

**Conclusion:**

Follow-up completeness is a pre-requisite for reliable outcome assessment and should be declared systematically. FUI represents a simple measure suited as reporting standard. Evidence lacking such information must be challenged as potentially flawed by selection bias.

## Introduction

Assessment of clinical outcomes and treatment efficacy depends on reliable follow-up information [1,2] Since aggregate evidence is at best as reliable as the underlying findings, unrecognized individual study flaws may eventually affect prioritization of research and development resources, regulatory processes and, ultimately, delivery of health care [3,4].

The completeness of follow-up is an important determinant of validity [5,6]. Clinical studies are expected implicitly to consider the course of all participants up to the “study end” [7]. Yet to avoid selection bias, specific start and end dates of the study must be pre-specified, declared and systematically applied. Kaplan-Meier analyses are widely used to adjust for variations in follow-up periods [8,9]; the associated extrapolations however, are only valid if these variations are non-selective [10]. Selectively recorded events, in contrast, may lead to relevant misestimations [6,11]. Thus of all potential flaws, incomplete follow-up is particularly dangerous as it may go unnoticed within flawed Kaplan-Meier estimates.

Ideally, study findings should be based on complete follow-up information [12]. But in reality, it may be impracticable to follow every single study participant exactly to the study end date. Therefore, studies should declare at least how complete their follow-up was, since otherwise their validity cannot be judged [13]. Nonetheless, none of the accepted reporting guidelines (eg. STROBE or CONSORT) currently calls for such declaration [12,14].

The present study evaluated the *Follow-Up Index* (FUI), a simple and flexible measure describing the actual follow-up period as a proportion of the actually possible follow-up period on an individual patient level. Given the hypothesis that unaccounted follow-up time correlates inversely with the accuracy of outcome estimates, the FUI could be expected to help evaluating the risk of selection bias and the credibility of study findings.

## Materials and Methods

**Fig 1:**
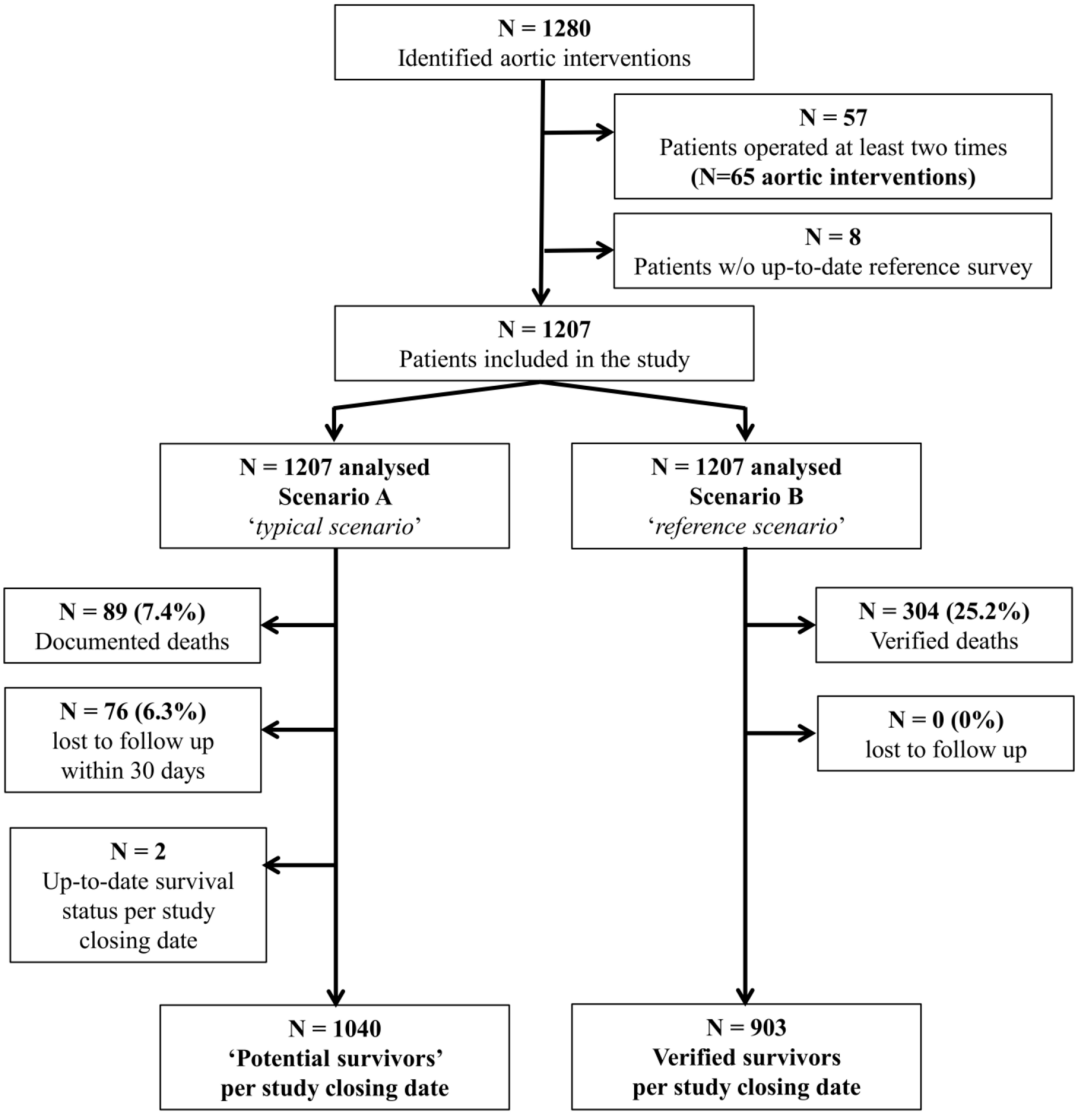
Patient flow through the study. Interventions were identified from a prospective registry of consecutive aortic interventions. Each patient was included only once (i.e., for the latest aortic intervention) during the study period.

The FUI was assessed in consecutive patients undergoing aortic repair during an 11 year period (June 2001 to December 2012) at a tertiary referral University hospital (Bern, Switzerland). The start of the study period was triggered by the hospital changeover to a SAP-based administration system (SAP ERP 6.06, SAP AG, Walldorf, Germany) that could be interrogated electronically. The study included a pilot and a completion phase, each with predefined study start and end dates. Cumulative long-term survival at the study end was calculated using Kaplan-Meier curves based on prospective registry information collected during clinical routine practice (Fig 1, **Scenario A**). This represents typical clinical (registry-based) outcome research. As control, Kaplan Meier curves were re-calculated after a comprehensive cross-sectional survey was conducted across the study population at the pre-specified study end date (**Scenario B**). Discrepancies between the two scenarios were evaluated regarding FUI, absolute follow-up periods, number of registered deaths and cumulative survival estimates. The predictive value of FUI was determined using multivariate correlation with the survival estimate discrepancy.

All patients gave written consent at the time of aortic repair to being further contacted during follow-up for clinical and scientific quality control, and the observational design of the study had been approved by the institutional research ethics committee. Data were analyzed anonymously. The report was prepared according to STROBE [12].

### Definitions

**Fig 2:**
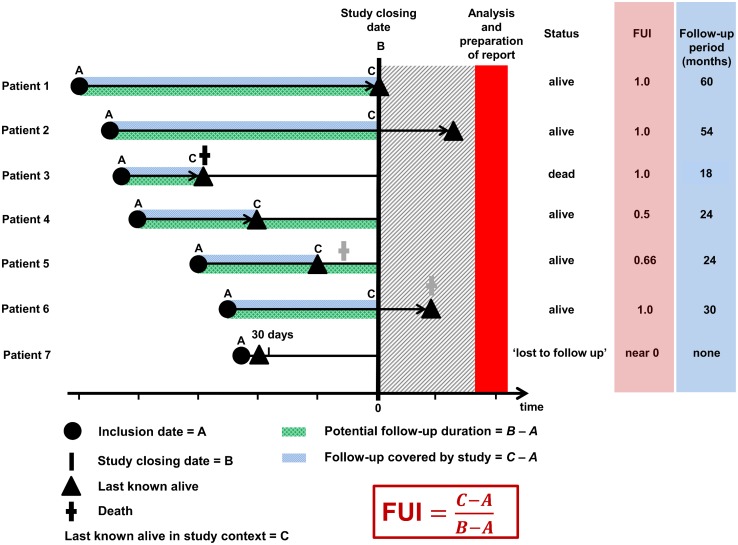
Proposed principle of follow-up assessment. Individual follow-up is characterized by two indicators: *absolute duration* and *completeness*. The *duration* measures the time, for which valid information on the investigated outcome is available (patients 1 to 6), but must end at the study closing date, even if information becomes available thereafter (patients 2 and 6). Similarly, clinical outcome is defined at this very closing date (patient 6). Summary statistics exclude those known to have died (patient 3) as well as those lost to follow-up within 30 days (patient 7). Both subgroups are reported separately as proportions; those who have died with a median time to death. The *completeness*, in contrast, is expressed as proportion (follow-up index, FUI), calculated as displayed. Patients known to be alive (patients 1 and 2) and patients known to be dead (patient 3) carry a FUI of 1 by default, all others have a FUI between 0 and 1. The unaccounted follow-up period (1 *minus* FUI) may hide events (patient 5) leading to underestimation bias. Therefore, the closer the FUI to 1 the smaller the risk of selection bias.

Follow-up periods were measured in days relative to the declared study end date (Fig 2). In each scenario, two distinct measures were calculated for each patient: (1) the absolute follow-up duration between the aortic intervention and the date ‘last known alive’; and (2) the FUI, defined as the ratio between the investigated follow-up period and the theoretically possible follow-up period up to the pre-specified study end date. As a proportion, FUI must range between 0 and 1: patients lost to follow-up directly after treatment would have a FUI near 0, whereas patients with follow-up to the study end date would have a FUI of 1 (Fig 2).

Patients known to have died during follow-up were declared as separate proportion with a median time to death. The term ‘lost to follow-up’ was limited to patients for whom the latest follow-up information lay within 30 days after aortic repair. This subgroup was also reported as separate proportion. Thus, the mean duration of follow-up was summarized from assumedly surviving patients with more than one month of follow-up. In patients undergoing more than one aortic intervention during the study period only the latest intervention was considered to avoid double entries. Lastly, patients for whom the actual survival information could not be ascertained eventually (i.e., within control scenario B) were excluded from analysis.

### Study cohort

The pilot phase involved patients undergoing open or endovascular repair of abdominal aortic aneurysm (AAA), whereas the completion study included patients undergoing repair of thoraco-abdominal aortic aneurysm (TAA). During analysis, both study populations were combined. To ascertain consecutive patient identification, all interventions were prospectively collected into a dedicated vascular surgery registry (Dendrite, version 1.6.8, Dendrite Clinical Systems Ltd, Henley-on-Thames, UK), which features patient and intervention related variables including age, sex, body mass index, cardiovascular risk factors, type of and indication for repair and date of intervention. Dedicated data managers were employed during the study period to scrutinize continuously all patient-related data whether they corresponded to clinical hospital notes to reproduce the actual base for clinical decision making. Missing values were completed as far as accordant information could be found in the clinical documentation, but no further examinations were performed to validate existing information on comorbidities.

### Follow-up assessment

Clinically, patients were followed according to standard in-house surveillance protocols (involving duplex scans every two years after open AAA repair and yearly imaging after endovascular AAA repair or any TAA repair). Thereby, practice varied to some extent according to the preference of the treating physician. Clinical follow-up information was fed prospectively into the vascular surgery registry.

For **scenario A**, ‘last-know-alive’ dates or, if available, dates of death were retrieved from the vascular surgery registry as well as hospital and outpatient records within the cardiovascular department. This information was supplemented by data obtained from the hospital SAP system, which documents administrative data across all hospital departments including in- and outpatient visits and notices of death. Thereby, patients were assumed alive at least until the latest registered personal contact or until positive information of patient death. Of note, this information was not necessarily up-to-date; therefore calculated FUI ranged between 0 and 1.

For **scenario B**, in contrast, three investigators (S.W., C.K. and C.T.) conducted a comprehensive up-to-date cross-sectional telephone survey at the pre-defined study end (±2 weeks). Thereby, patients, relatives, family doctors or local authorities were contacted. Eventually, follow-up was complete in all patients; therefore calculated FUI was 1 for all. In both scenarios, follow-up information was compiled in a blinded fashion.

### Statistical methods

Time periods were reported as months. Conventional descriptive summary statistics were used for distributions and proportions (i.e, mean±standard deviation (SD) or median with interquartile range (iqr); and percentages, respectively). Follow-up scenarios (A vs B) were compared using FUI, absolute follow-up duration, numbers and proportions of discovered deaths and cumulative survival estimates as dependent variables. Differences were considered statistically significant at an alpha level of 0.05. All tests were two-sided and paired, and in general non-parametric tests were used.

Cumulative long-term survival was estimated for each scenario separately by Kaplan-Meier method [10]: **Scenario A** considered patients up to the ‘last-known-alive’ date (Fig 2). Thereafter, patients were either uncensored (if they had died at this date) or censored (if no further information was available). **Scenario B**, in contrast, did not censor patients, since survival status or date of death was known for each patient up to the study end date. In either scenario, events occurring after the study end date were ignored (Fig 2). To account for matched pairs between scenarios, survival estimates were compared using a multivariable Cox regression mixed-effects model (anonymized follow-up information according to scenario in S1 Minimal Dataset).

Obviously, any discrepancy between the curves can only be produced by ‘potential survivors’ in scenario A (i.e., those with a FUI<1, Fig 1), because all other patients are equally known to be either dead or alive at the study end in both scenarios (i.e., those with a FUI = 1, Fig 2). Therefore, the potential survivors were further investigated to determine to which extent FUI correlated with the accuracy of cumulative survival estimates: **Scenario A** attributed them a constant survival estimate of 100% over time, since it had no knowledge of any death in these patients. In contrast, **scenario B** estimated the mortality rate in the same patients based on actual survival information. Therefore, any difference between scenario A (0% mortality) and scenario B measures directly by how much scenario A underestimated mortality over time.

According to study hypothesis, mortality underestimates are expected to increase with decreasing FUI (i.e. increasing lack of follow-up information). This hypothesis was tested using FUI quartiles (quartile 1 with the highest FUI values down to quartile 4 with the lowest FUI values), which were entered as predictor variable into a scenario-stratified cox proportional hazards model analysing four equally sized groups. The observed discrepancy between mortality estimates served as outcome variable. In a primary adjusted model patient age and sex, type of repair and the time since treatment were suspected confounding factors and entered as covariates for adjustment. None of the values was missing; therefore none of the participants had to be excluded from multivariate analysis. In a secondary adjusted model comorbidities (coronary heart disease, chronic obstructive lung disease, dyslipidemia, diabetes, arterial hypertension, smoking status and renal insufficiency) were considered additionally. In an analogous approach, the predictor (FUI) was grouped into 10 ordinal categories by fixed intervals (0.0–0.09; 0.10–0.19; etc). Effects of increasing FUI quartiles and ordinal categories, respectively, were reported as scenario-stratified adjusted hazard ratio (HR) with 95% confidence intervals (CI). Lastly, in a sensitivity analysis, the subsets of patients with complete follow-up information at one, two and three years (scenario A) were selectively evaluated and compared to the (assumedly correct) survival estimates among the whole study population in scenario B. STATA 12 (StataCorp, LP, Texas, United States) and IBM SPSS for Windows (Version 21.0, Armonk, New York) were used for all statistical calculations.

## Results

Overall, 1280 aortic interventions were registered during the study period (n = 769 (63.7%) for AAA; and n = 438 (36.3%) for TAA, respectively). In 65 patients undergoing a repeat aortic intervention during the study period, only the latter was included. In addition, 8 patients were excluded because they could not be reached eventually during the reference survey (scenario B). Thus, 1207 interventions were analysed according to study protocol (Fig 1). The theoretical minimum follow-up duration was 4 months, whilst the theoretical maximum was 130 months. Patient and intervention-related characteristics are summarized in Table 1.

**Table 1:** Patient and intervention-related characteristics.

	Study cohort
	n = 1207
Male sex, *n*	1028 (85.2%)
Age in years	70 (65; 77)
Body mass index in *kg/m* ^*2*^	26.8 (24.6; 31.3)
missing information, *n*	120 (9.9%)
Operated at least two times, *n*	57 (4.7%)
**Comorbidities and surgical risk factors**	
Coronary artery disease, *n*	537 (44.5%)
missing information	8 (0.7%)
Arterial hypertension, *n*	1018 (84.3%)
missing information	6 (0.5%)
Current smoker, *n*, yes/*never*	432 (35.8%) / 322 (26.7%)
ex-smoker	442 (36.6%)
missing information	11 (0.9%)
Chronic obstructive lung disease, *n*	256 (21.1%)
missing information	6 (0.5%)
Diabetes mellitus, *n*, yes/no	167 (13.9%)
missing information	8 (0.7%)
Renal insufficiency, *n*	230 (19.1%)
missing information	6 (0.5%)
Dyslipidemia, *n*	745 (61.7%)
missing information	9 (0.7%)
**Intervention-related characteristics**	
Abdominal aortic aneurysm repair, *n*	769 (63.7%)
Endovascular repair, *n*	341 (28.3%)

Summary statistics are given as absolute numbers (%) or as median (interquartile range).

**Fig 3:**
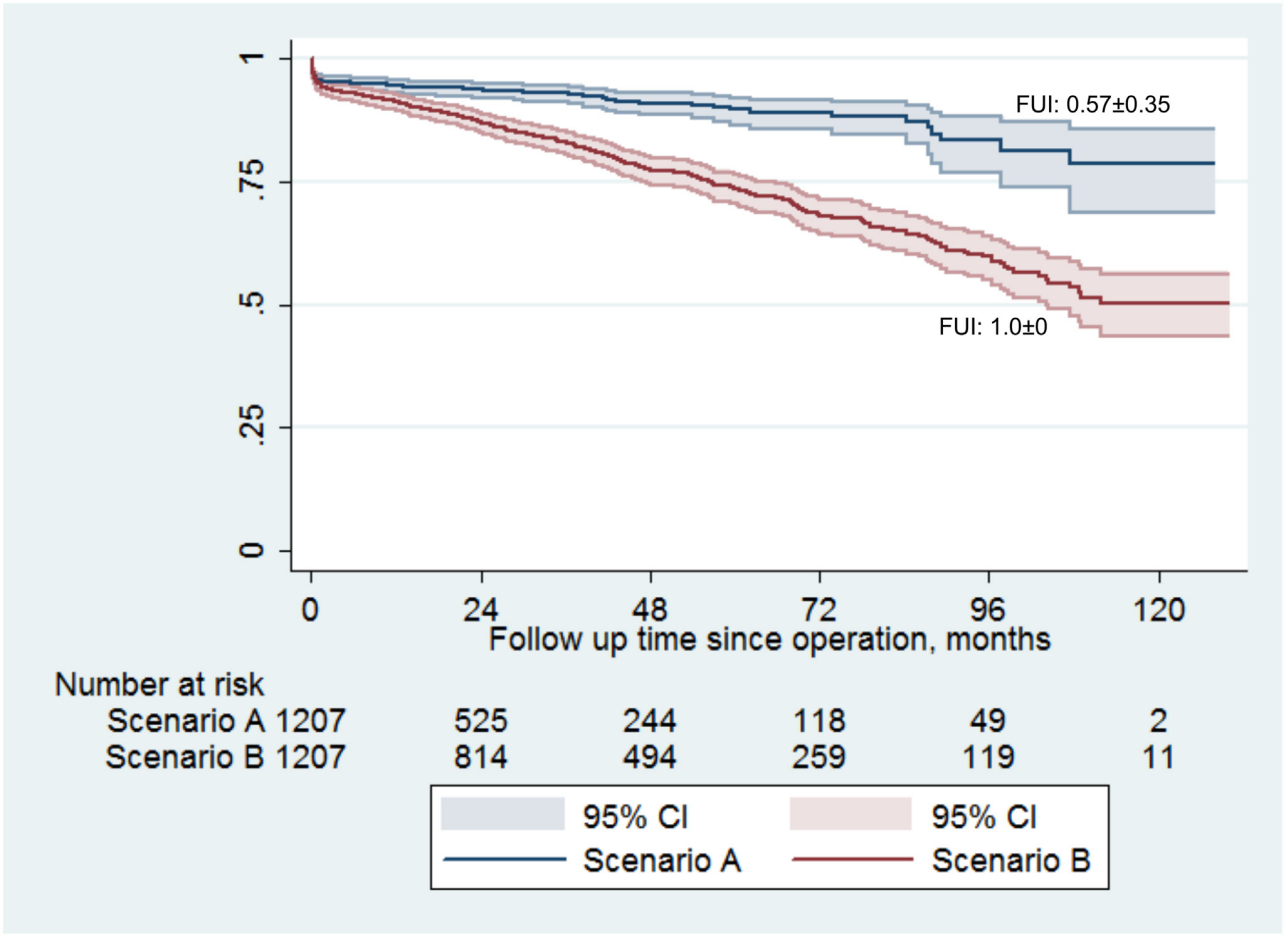
Kaplan Meier long-term survival estimates for the study population (n = 1207) according to completeness of follow-up. Scenario A (blue curve) estimated survival based on registry data, which, although collected prospectively during clinical routine, were not up to date for every patient at the study end. Scenario B (red curve), however, estimated survival of the same study population based on a comprehensive survey performed at the study end. Completeness of follow-up differed significantly between scenarios as expressed as follow-up index (FUI, see text). Thereby, scenario A (FUI 0.57±0.35) underestimated effective mortality by almost 30% (scenario B; FUI 1.0±0).

**Fig 4:**
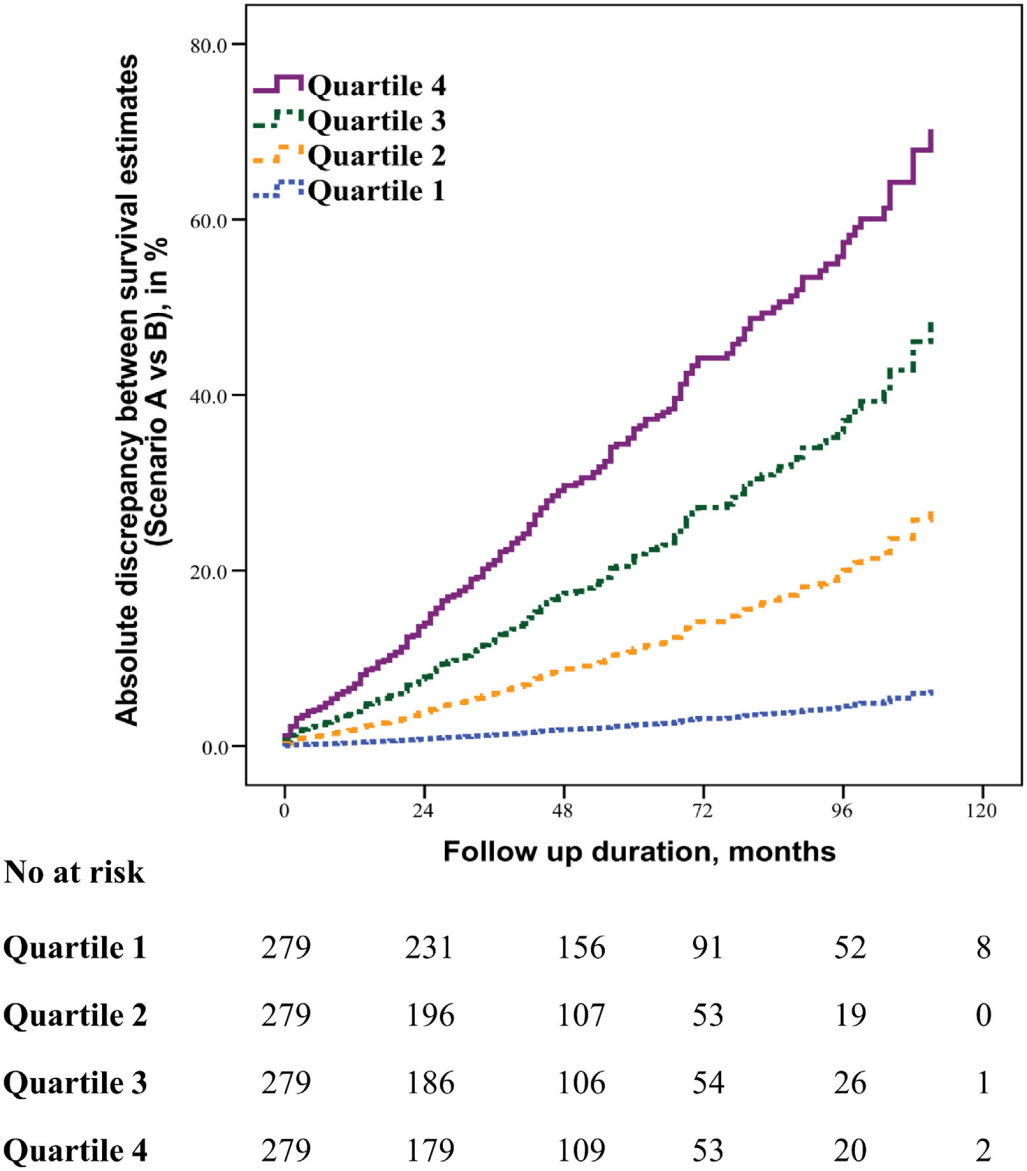
Association between follow-up index (FUI) and the degree of underestimated mortality among ‘potential survivors’ (n = 1116). Patients were grouped into equally sized quartiles according to FUI (quartile 1 with highest FUIs; quartile 4 with lowest FUIs). After adjustment for potential confounding factors, underestimation of the actual mortality (i.e., inaccuracy of outcome estimate) correlated significantly with decreasing completeness of follow-up (see Table 2).

In **scenario A**, prospective clinical routine patient documentation covered an absolute follow-up period of 30±28 months, corresponding to a mean FUI of 0.57±0.35 relative to the pre-defined study end date. 76 patients had been lost to follow-up within 30 days after aortic repair (6.3%); and 89 deaths (7.4%) were known to have occurred after a median time to death of 0.5 months (iqr 0.1; 22.5, FUI = 1). Two patients were actually in hospital at the study closing date (FUI = 1). Thus, a total of 1116 patient were “potential survivors” with a FUI<1 in scenario A. In contrast, survival status at the study end date (±2 weeks) was authenticated for all 1207 patients in **scenario B**. Thus, it evaluated the complete follow-up period of 49±32 months (*P*<0.001) corresponding to a mean FUI of 1.0±0.0 (*P*<0.001). Scenario B brought forward a total of 304 actual deaths (25.2%, as compared to n = 89 in scenario A, *P*<0.001) after a median of 25.5 months (iqr 2.8; 54.5; *P*<0.001). The discrepancy of 215 deaths impacted long-term survival estimates significantly (Fig 3): scenario A postulated 78.7% survival at the end of follow-up, whereas scenario B showed only 50.7% survivors (Cox regression mixed-effects model, *P*<0.001). As hypothesised, the discrepancy between survival estimates correlated with decreasing FUI-quartiles (Fig 4 and Table 2) as well as with decreasing FUI-intervals: underestimation of long-term mortality increased almost linearly by 30% with each 0.1 drop in FUI (adjusted HR 1.30; 95%-CI 1.24, 1.36; P<0.001). These effects were all independent of patient age and sex, duration of follow-up, year of intervention, surgical management and study phase. They were also unaffected by patient comorbidities (unchanged findings in the secondary adjusted model (Table 2)).

**Table 2:** Association between FUI and underestimation of mortality in potential survivors (n = 1116).

	Mean FUI (±SD)	Missed events, n	Underestimation of mortality compared to complete follow-up (per cent)	Hazard Ratio (95% CI)		
				Unadjusted	*Adjusted	* *Adjusted P Value*
**FUI-quartile 1** (n = 279)	0.94 ± 0.04	8	**11.1**	1.00 (reference)	1.00 (reference)	n/a
**FUI-quartile 2** (n = 279)	0.73 ± 0.07	31	**50.4**	5.44 (2.5; 11.9)	4.81 (2.2; 10.5)	<0.001
**FUI-quartile 3** (n = 279)	0.39 ± 0.12	65	**59.6**	11.47 (5.5; 23.9)	10.0 (4.8; 20.8)	<0.001
**FUI-quartile 4** (n = 279)	0.06 ± 0.05	111	**62.3**	20.16 (9.8; 41.4)	18.38 (8.9; 38.0)	<0.001

* Hazard ratios (stratified by *scenario*), confidence intervals and *P*-values were adjusted for baseline age, sex, time since operation, type of operation (AAA repair vs TAA repair, endovascular repair vs open repair) in a primary adjusted model. In a secondary adjusted model comorbidities (coronary heart disease, diabetes, renal insufficiency, chronic pulmonary lung disease, smoking status, arterial hypertension, dyslipidemia) were considered additionally, but this did not alter the findings of the primary adjusted model

CI, Confidence Interval; SD, standard deviation

The sensitivity analyses based on samples with complete 1, 2 or 3-year follow-up in scenario A (i.e., each with a mean FUI of 1) included 786 patients (65.1%), 593 patients (49.1%) and 427 patients (35.4%), respectively. The actual survival rates (92.0% (95%-CI: 90.1; 93.9); 88.5% (85.9; 91.1) and 83.4% (79.9; 86.9), respectively) in these scenario A subsets were almost identical to the actual mortality rates within the whole study population (scenario B): 91.2% (n = 1037 at risk) at 1 year; 87.0% (n = 814 at risk) at 2 years; and 83.0% (n = 647 at risk) at 3 years.

## Discussion

In contrast to other methodological challenges [15], selection bias introduced by incomplete follow-up is rarely appreciated [2]. The present study demonstrated how easily significant proportions of follow-up are missed unconsciously in typical clinical reports (**scenario A**), and what discrepancies may result if the same patient sample was scrutinized thoroughly over the same study period again. The fundamental finding was not the absolute size of the misestimation but that it would remain completely unsuspected even if the report followed all current reporting standards (eg. STROBE or CONSORT) [12,14]. These standards seem to ignore that reliability of individual study findings cannot be appreciated without a suitable measure of follow-up completeness, implying that the current body of evidence (which did not declare whether every single patient was followed up to a prespecified study end date) might be based on flawed assumptions.

It is easily forgotten that reliable outcome assessment depends on whether or not the study end has been defined upfront, because any post hoc inclusion or (unconscious) exclusion of outcome events will lead to selection bias [6,9,11,14]. Under this premise only, variable follow-up periods may be subsumed as Kaplan Meier curves. But authors, reviewers and readers have become so accustomed to survival curves that the consequences of not taking into account missed follow-up periods remain uncritically ignored. This study demonstrated that indeed this may be clinically important.

The present observations are only relevant if they represent typical hazards of outcome studies. Considering that the present study underestimated the actual mortality by 30% (Fig 3), one could presume an exceptionally poor clinical follow-up. However, patients were enrolled and followed prospectively according to clinical guidelines, and hospital-wide, not only departmental, administrative data were interrogated for death notices. Even such unusual efforts towards comprehensive follow-up did not translate into coverage of more than 60% follow-up time at the given study date (i.e. FUI of 0.57). In a similar study, Jensen and colleagues compared mortality extrapolations from a clinically fed registry to independently updated survival information [8]. Among 102 vascular patients, they found a 10% discrepancy between survival estimates already at one year, which is even larger than in the present study. Clark and colleagues used the ‘completeness-index C’ to measure follow-up completeness of several large prospective cohort studies and randomized trials [2]. Thereby, the ratios between the summed-up observed versus the summed-up potential follow-up times were calculated across whole study groups. They found that even under optimally controlled study conditions, overall follow-up completeness ranged as low as 69%. Thus, both studies imply that the present example reflects clinical research realistically. Of note, neither explored the relationship between follow-up completeness and accuracy of study findings. That the conceptually convincing C-index did not prevail is probably due to its complexity, statistical inflexibility and undefined predictive value and emphasises the need for a practical indicator [13].

There are established indicators such as the mean follow-up duration or the proportion of those ‘lost to follow-up’ [9,14]. However, neither of these indicators considers unaccounted follow-up time, neither has been uniformly defined [16,17] and none has been shown to correlate with outcome accuracy. In contrast, the FUI expands the concept of the C-index [2] to an individual level which offers several important advantages (Fig 2): the FUI is clearly defined by three individual dates that are easily available for every patient in any serious outcome research (i.e. date of inclusion/treatment, date of last contact and study end date). It complements the declared summary follow-up duration and takes into account those lost to follow-up, thereby eliminating an ambiguous parameter and standardising reports (Fig 2). But most importantly, it describes the individual distribution of follow-up completeness between study participants thereby offering the opportunity for stratification and multivariable adjustments. Last but not least, considering that both, FUI quartiles and FUI intervals correlated almost linearly with the accuracy of survival estimates (Fig 4 and Table 2), it helps critically appraising study credibility.

Thereby, interpretation of the FUI is less straightforward than it may appear at first sight and resembles that of the *P*-value in many ways [18]. Most importantly FUI indexes only a probability (i.e. the risk of unreliable study results occurring), but not the actual size or clinical significance of any aberration. The latter is primarily a function of the investigated outcome and its natural incidence within the study population. For instance, among 70 year old vascular patients, each 0.1 drop in FUI after aortic aneurysm repair reduced the accuracy of the reported mortality 1.3 fold. This flaw would probably be much smaller in healthier populations with a lower natural incidence of death, for instance in 35 year old patients after appendectomy. Therefore, FUI is an indirect contextual measure which makes a universal FUI-threshold for ‘outcome credibility’ unlikely to be defined. Future studies may use statistical simulations to define meaningful FUI cut-off values for specific patient populations, surveillance programmes or particular outcomes [19]. But even then, knowledge of FUI will not safeguard against misinterpretation due to other flaws [15].

Trust into scientific integrity has been the traditional mainstay of clinical research. The increased awareness of breaches and study retraction rates [20–22] has led to the establishment of quality assurance initiatives and best practice guidelines [23–25]. The FUI may be seen in this context. Particularly in retrospective studies or post-hoc analyses, authors provide only rarely details about the quality of data acquisition. As long as standardized disclosure of follow-up completeness (as, for instance, in Fig 2) is not mandatory [12,14], a fine line will persist between fraud on one hand and unintentional reporting of inaccurate outcomes on the other [23]. Increasing awareness and a suitable measure would both challenge ignorance as an acceptable excuse for publishing misleading results. Importantly, these considerations apply independently of the study design, i.e. just as well to randomized trials [2,4]: although randomization may balance patient-related factors between trial arms before the intervention effectively, it cannot protect from disparities (and biased findings) between trial arms during follow-up [26]. Thus randomized trials should not only disclose baseline characteristics and absolute follow-up duration to demonstrate comparability of study groups but also their follow-up completeness (i.e. FUI).

The FUI has only been evaluated in a specific patient population from a single centre that was prospectively recorded mainly for clinical quality assurance purposes. Impact of comorbidities, treatment strategy, postoperative surveillance programme and study era was only assessed within these limitations. Thereby, some information on specific patient characteristics was missing (Table 1) possibly influencing multivariable adjustments. But proportions of missing values were small and patients were combined in separate categories during multivariate analyses. Even though the hypothesized correlation between gaps in follow-up and accuracy of outcome estimates seems generally plausible, mathematically obvious and was unaffected by potential confounding factors in this homogeneous sample, external validation in larger, preferably population based patient samples is needed.

Complete follow-up information of every single patient will probably remain an unrealistic goal for most clinical research, but at the very least completeness should be declared. Based on the present observations, every effort should be made to approach the ideal of a mean FUI of 1.0, even if this leads to seemingly worse outcomes than in previous (possibly biased) reports. Therefore, feasible strategies for effective cross-sectional outcome assessment will have to be evaluated, particularly in large patient populations.

To conclude, the present findings challenge the existing body of clinical evidence by highlighting the critical relevance of follow-up completeness, which is largely ignored in the literature. In the future, transparent declaration of follow-up completeness should be demanded systematically for all types of clinical studies to enable critical appraisal. The FUI is proposed as a simple, readily available, versatile and highly predictive standard indicator of the credibility of study findings.

## Supporting Information

S1 Minimal Dataset Dataset containing anonymized follow-up information. Follow-up was collected twice for the same cohort using two different approaches (scenario A and scenario B, presented as access database).
(ACCDB)
